# Ploidy Testing of Blastocoel Fluid for Screening May Be Technically Challenging and More Invasive Than That of Spent Cell Culture Media

**DOI:** 10.3389/fphys.2022.794210

**Published:** 2022-02-21

**Authors:** Wenhao Shi, Zhenghao Zhao, Xia Xue, Qian Li, Yaxin Yao, Dongyang Wang, Jing Wang, Sijia Lu, Juanzi Shi

**Affiliations:** ^1^The Assisted Reproduction Center, Northwest Women’s and Children’s Hospital, Xi’an, China; ^2^Department of Clinical Research, Yikon Genomics Company, Ltd., Suzhou, China; ^3^Translational Medicine Center, Northwest Women’s and Children’s Hospital, Xi’an, China

**Keywords:** pre-implantation genetic testing of aneuploidies, inner cell mass, spent culture medium, blastocoel fluid, embryos

## Abstract

**Background:**

Recent studies have demonstrated that both blastocoel fluid (BF) and spent cell culture media (SCM) have potential as materials for non-invasive or less-invasive pre-implantation genetic analysis. BF may allow more opportunity to obtain cell-free DNA from the inner cell mass (ICM), and it has a lower risk of containing contaminant DNA from cumulus cells, sperm and culture media. There are no data regarding the ICM as a gold standard to evaluate the chromosome constitution of BF or SCM for embryo liquid biopsy.

**Methods:**

Two hundred eighteen donated human blastocysts were warmed and cultured in blastocyst culture media for 18–24 h. The corresponding SCM was collected, and only clear ICM was biopsied in blastocysts; otherwise, the whole blastocyst (WB) was biopsied. Quantitative PCR was performed to determine the DNA levels in the SCM and BF before and after amplification. ChromInst was used to amplify BF/SCM and blastocyst DNA before sequencing. Chromosomal copy number variation (CNV) was investigated to evaluate the chromosome constitution.

**Results:**

In total, 212 blastocysts were available for SCM and BF collection. The technical success rates (next-generation sequencing data) were 100 and 69.8% (148/212) for SCM and BF, respectively. Among the 148 blastocysts with both SCM and BF data, 101 were euploid and 47 were aneuploid based on ICM (*n* = 89) or WB (*n* = 59) analysis as the gold standard. Among all blastocysts, SCM was comparable to BF [specificity: 80.2 versus 61.4% (*P* = 0.005, χ^2^ test); sensitivity: 91.5 versus 87.2% (*P* = 0.738, χ^2^ test); negative predictive value (NPV): 95.3 versus 91.2% (*P* = 0.487, χ^2^ test); positive predictive value (PPV): 68.3% versus 51.3% (*P* = 0.042, χ^2^ test)]. The SCM and BF samples were 83.8% (124/148) and 69.6% (103/148) concordant with the corresponding ICM/WB samples when only two categories, euploid or aneuploid/mosaic, were grouped to calculate the concordance.

**Conclusions:**

Compared with BF, SCM has superior diagnostic performance, and it is non-invasive for embryos.

**Clinical Trial Registration:**

[http://www.chictr.org.cn], identifier [ChiCTR-BPD-17014087].

## Introduction

Aneuploidy in human embryos is associated with unfavorable reproductive outcomes, including implantation failure, pregnancy loss, and birth defects (Rubio et al., 2005; Fragouli and Wells, 2012; Campos-Galindo et al., 2015). Pre-implantation genetic testing of aneuploidies (PGT-A) in *in vitro* fertilization (IVF) treatment allows the selection of euploid embryos for transfer, thereby improving pregnancy outcomes (Sullivan-Pyke and Dokras, 2018). Conventional PGT-A is cell-based but adversely impacts embryo development (Mastenbroek et al., 2011). Although the long-term effect of embryo biopsy has not been investigated in humans, investigators have observed that embryo biopsy negatively influences neural and adrenal development in mice (Zeng et al., 2013; Wu et al., 2014). Additionally, embryo biopsy requires sophisticated equipment and extensive expertise. Therefore, a non-invasive and convenient PGT-A approach may have better acceptability and broaden the application of PGT-A before embryo implantation, thereby improving the success rates of clinical IVF.

Recent studies have demonstrated that both blastocoel fluid (BF) and spent culture medium (SCM) contain genetic material, which could be used for PGT-A (Gianaroli et al., 2014; Xu et al., 2016; Vera-Rodriguez et al., 2018). However, the concordance rate of the two DNA sources to the gold standards obtained with trophectoderm biopsy or the whole embryo varies significantly in different publications. Blastocentesis is required to obtain BF but has a high failure rate. Furthermore, the proportion of successfully performed whole-genome amplification (WGA) and production of detectable levels of DNA ranges from 34.8 to 82%, with a concordance rate varying between 37.5 and 97.4% (Palini et al., 2013; Gianaroli et al., 2014; Tobler et al., 2015; Magli et al., 2016; Zhang et al., 2016; Magli et al., 2019).

Due to different sampling methods and the “gold standard,” substantial variation from 33 to 100% has been observed in the chromosomal ploidy consistency of cell-free DNA in SCM with trophectoderm biopsy or whole blastocyst (WB) biopsy, and there is a broad variation from 77.3 to 100% in the success rate of WGA of SCM (Shamonki et al., 2016; Kuznyetsov et al., 2018; Vera-Rodriguez et al., 2018; Rubio et al., 2019; Yeung et al., 2019). These wide variations in results regarding chromosomal constitutional concordance indicate that a high-quality and comprehensive comparative study of SCM and BF for selecting euploid embryos should be performed to address the inconsistencies.

In the current study, we used 218 embryos obtained via IVF to compare the diagnostic performance of BF to that of SCM as the source material for non-invasive chromosome ploidy testing of embryos, with the WB and the ICM as references.

## Materials and Methods

### Ethics Statement

The study protocol was approved by the Ethics Committee of Northwest Women’s and Children’s Hospital, Xian, China (No. 2017121101). The study is registered at the Chinese Clinical Trial Registry (ChiCTR-BPD-17014087).^1^ All embryos were donated from women who received IVF or intracytoplasmic sperm injection (ICSI) and had surplus embryos preserved after giving birth to live healthy babies. Informed consent was obtained for each embryo sample used in this study.

### Embryo Culture and Blastocyst Cryopreservation

Standard protocols were used for ICSI or IVF. Embryos were cultured individually in 25-μL microdrops of global total with human serum albumin (HSA) (LifeGlobal) overlain with mineral oil in Miri incubators (ESCO) at 37°C in a dry atmosphere of 5% O_2_, 6–7% CO_2_ balanced with N_2_. Embryos did not undergo any extra manipulation during the culture period before biopsy, i.e., no zona drilling on day 3. Embryos were evaluated on day 5 or on day 6. Morphology assessment encompassed developmental stage, blastocyst expansion, and quality of ICM and TE. Blastocysts were graded using published criteria1. A single blastocyst was selected and transferred. While the remained embryo was cryopreserved by vitrification (Vit Kit–Freeze, IrvineScientific, Santa Ana, CA, United States). Before cryopreservation, the blastocysts were treated with a laser to induce artificial shrinkage. Women who had surplus embryos preserved after giving birth to live healthy babies donated embryos. And all those embryos used for subsequent analysis.

### Blastocentesis, Spent Cell Culture Media Collection and Blastocyst Biopsy

**FIGURE 1:**
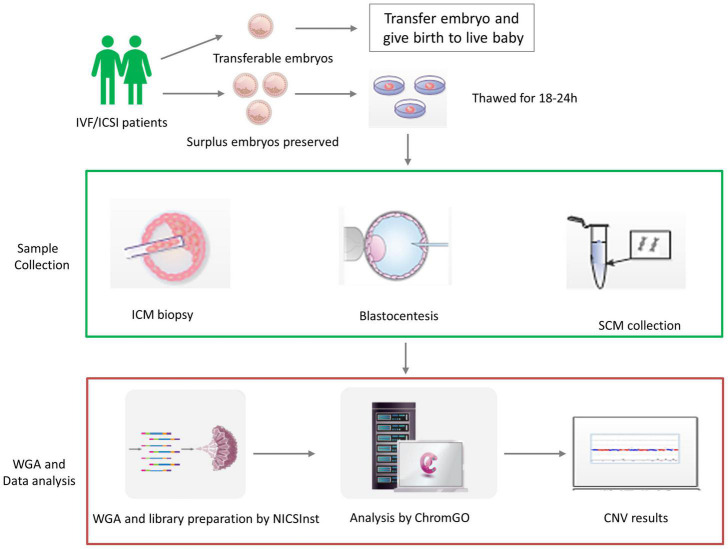
The validation procedure of the study design. Human blastocysts were donated by *in vitro* fertilization (IVF)/intracytoplasmic sperm injection (ICSI) patients with preserved surplus embryos after giving birth to live healthy babies. We then validated non-invasive chromosome screening (NICS) using spent culture medium (SCM) versus blastocoel fluid (BF) as a source of DNA. In each embryo, we obtained the chromosome ploidy results from the inner cell mass (ICM), trophectoderm biopsy (TE), SCM, and BF after whole-genome amplification (WGA) and library preparation by NICSInst. Copy number variation (CNV) was determined by ChromGo Analysis Software.

The flowchart of sample collection is shown in Figure 1, and the blastocentesis and ICM biopsy are shown in Supplementary Figure 1. Two hundred eighteen human blastocysts donated by 40 couples were used. Vitrified blastocysts were thawed and washed extensively with G-2 blastocyst culture medium (G5 Series, Vitrolife, Gothenburg, Sweden) supplemented with 10% human serum albumin (HSA) and cultured in 20-μL medium for 18–24 h until the blastocysts became fully expanded. SCM was collected into an RNase–DNase-free PCR tube containing 5 μL cell lysis buffer (Yikon Genomics, Suzhou, China). Blastocentesis was carried out when the blastocyst was fully re-expanded as previously reported (Tobler et al., 2015). In addition, SCM was collected simultaneously. Culture medium (20 μL) from the same batch but free of contact with embryos was included as the background control. All samples were snap frozen and stored at −80°C prior to analysis.

After blastocentesis, the blastocyst was cultured for another 4–10 h for re-expansion prior to ICM biopsy and collection of the WB. ICM biopsy was conducted as described previously (Capalbo et al., 2013) in fully re-expanded blastocysts (expansion grades 4–6) with grade A/B morphology only, and the WB, whole blastocyst with a clearly visible ICM, was biopsied. The biopsy was performed in dishes prepared with three droplets (10-μL G-MOPS, G5 Series, Vitrolife) overlaid with oil (Vitrolife) equipped with micromanipulation and a pulsed-field laser. A laser was used to assist the breaching of a 20-μmm hole in the zona pellucida. The ICM was aspirated using a standard blastomere biopsy pipette (30 μm, 30^°^, Sunlight Medical) by introducing the biopsy pipette directly into the blastocoel cavity and pushing in and stretching the trophectoderm layer mechanically. The ICM was aspirated and removed from the embryo with the assistance of laser and mechanical cutting. The video of ICM biopsy and the images of fluorescence staining of the ICM tissue are shown in Supplementary Figure 2.

### Next Generation Sequencing

Single-cell WGA was conducted using library preparation by ChromInst (EK100100724 NICSInst™ Library Preparation Kit, Yikon Genomics). Sequencing was conducted using an Illumina MiSeq platform, yielding ∼2 million sequencing reads on each sample (Fang et al., 2019). The Next Generation Sequencing (NGS) data were analyzed, and chromosomal copy number variation (CNV) was investigated as described previously (Huang et al., 2015; Xu et al., 2016; Shang et al., 2018). The numbers of high-quality reads were counted along the whole genome with a bin size of 1 Mb and normalized by the GC content and a reference dataset. The circular binary segmentation (CBS) algorithm was used to detect CNV segments. A copy number gain from two to three copies results in a 50% increase in read counts, whereas a copy number loss from two copies to one copy results in a 50% decrease in read counts. Aneuploid mosaicism above 50% was reported. CNV was reported by ChromGo^®^ Analysis Software (EK1001013, Yikon Genomics), and reports for non-invasive chromosome screening (NICS) were generated. Technical success was defined as the generation of reliable NGS data.

### Determination of DNA Content

A volume of 20 μL was used to determine the DNA concentration in each group. To exclude DNA contamination from the culture medium itself, DNA in HSA-free G2.5 and G2.5 containing 10% HSA was measured before and after WGA. Quantitative PCR (qPCR) was performed using specific primers for the multicopy-gene *duf1220* and SYBR Green master mix (BioRad, California, United States). PCR was run at 95°C for 10 min followed by 45 cycles of 95°C for 10 s and 60°C for 1 min.

### Statistical Analysis

Data analyses were conducted using SPSS version 19 (IBM, Armonk, NY, United States). Continuous variables were analyzed using Student’s *t-*test if normally distributed (as verified by the Shapiro–Wilk test) or the Mann–Whitney *U*-test if non-normally distributed. The main outcome measures of the study were specificity [specificity = (true negatives)/(true negatives + false positives)], sensitivity [sensitivity = (true positives)/(true positives + false negatives)], negative predictive value [NPV = (true negatives)/(true negatives + false negatives)] and positive predictive value [PPV = (true positives)/(true positives + false positives)] for ploidy and concordance rate for karyotype. Categorical variables were analyzed with the χ^2^ test. *P* < 0.05 (2-sided) was considered statistically significant.

## Results

### DNA Content and Karyotyping Success Rate With Spent Cell Culture Media Versus Blastocoel Fluid

**FIGURE 2:**
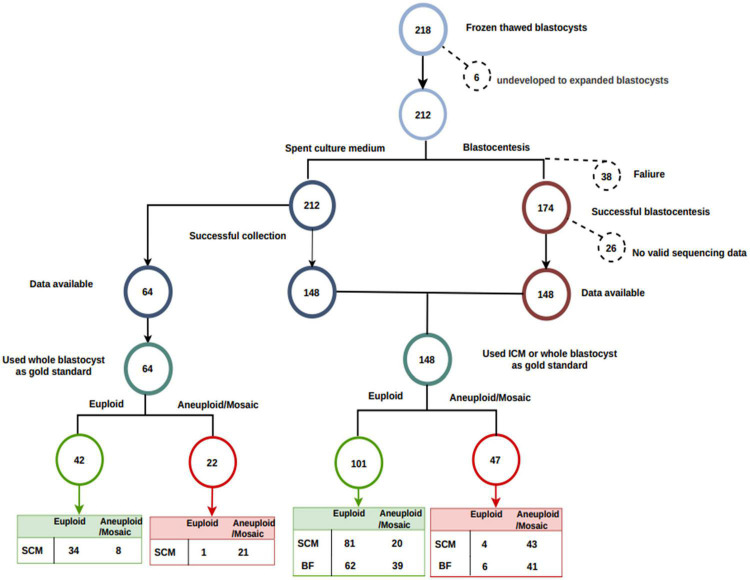
Flowchart of sample processing success and loss. In total, 212 of 218 blastocysts were fully re-expanded. Blastocentesis failed in 38 full-expanded blastocysts. No valid sequencing data (low reads number) were generated in an additional 26 blastocysts. Inner cell mass was available as a reference for 89 blastocysts, and whole embryo was used as a reference for the remaining 59 blastocysts. Among the 148 blastocysts with both SCM and BF data, 101 were euploid, and the remaining 47 were aneuploid.

Two hundred eighteen human blastocysts were donated for this study. We excluded six blastocysts that were not fully re-expanded and 38 blastocysts that failed blastocentesis. We also excluded 26 blastocysts with a low reads number (Figure 2). ICM was available as a reference for 89 blastocysts, and whole embryos were available as a reference for the remaining 59 blastocysts.

The median DNA concentration was 9.17 × 10^–6^ ng/μL (range 3.33–25.34 × 10^–6^ ng/μL) in HSA-free G2.5 prior to WGA and 1.32 ng/μL (range 0.37–1.95 ng/μL) following WGA and 14.6 × 10^–6^ ng/μL (range 5.20–61.00 × 10^–6^ ng/μL) in G2.5 containing 10% HSA prior to WGA and 1.12 ng/μL (range 0.35–2.08 ng/μL) following WGA (Supplementary Figure 3A). The median concentration of total DNA in SCM was 1.71 × 10^–3^ ng/μL (range 0.09–11.41 × 10^–3^ ng/μL) prior to WGA and 41.2 ng/μL (range 4.78–106 ng/μL) following WGA. In addition, the median concentration of total DNA in the BF was 0.31 × 10^–3^ ng/μL (range 0.03–2.58 × 10^–3^ ng/μL) before WGA and 14.8 ng/μL (range 0.812–62.2 ng/μL) following WGA. The median concentration of total DNA in SCM was significantly higher in SCM than in the BF both prior to and following WGA (*P* < 0.001, Mann–Whitney test) (Supplementary Figures 3A,B). DNA sequencing of 15 medium controls with DNA concentrations >1.5 ng/μL after WGA failed to yield valid sequencing data, while 100% (212/212) of SCM samples and 69.8% (148/212) of BF samples generated qualified NGS data (Supplementary Figure 3C). The findings indicated that SCM samples had a higher DNA content and sequencing success rate than BF samples.

### Diagnostic Performance of Karyotyping by Spent Cell Culture Media Versus Blastocoel Fluid

Inner cell mass or WB was used as the gold standard, 68.2% (101/148) of blastocysts were euploid, and 31.8% (47/148) were aneuploid/mosaic. Our analysis of 148 triads of WB/ICM-SCM-BF samples revealed that the specificity was 80.2% for SCM and 61.4% for BF (*P* = 0.005, χ^2^ test), and the sensitivity was 91.5% for SCM and 87.2% for BF (*P* = 0.738, χ^2^ test) (Table 1). The NPV was 95.3% for SCM and 91.2% for BF (*P* = 0.487, χ^2^test), and the PPV was 68.3% for SCM and 51.3% for BF (*P* = 0.042, χ^2^ test). SCM and BF samples were 83.8% (124/148) and 69.6% (103/148) concordant with the corresponding ICM/WB samples if only two categories, euploid or aneuploid/mosaic, were grouped to calculate the concordance.

**TABLE 1:** The diagnostic performance of NGS of spent culture medium and blastocoel fluid DNA for aneuploidy/mosaicism.

	Total			Consistency with inner cell mass			Consistency with whole blastocyst		
Assay	SCM	BF	*P*	SCM	BF	*P*	SCM	BF	*P*
Specificity	80.2% (81/101)	61.4% (62/101)	0.005	85.0% (51/60)	60.0% (36/60)	0.004	73.2% (30/41)	63.4% (26/41)	0.476
Sensitivity	91.5% (43/47)	87.2% (41/47)	0.738	93.1% (27/29)	86.2% (25/29)	0.670	88.9% (16/18)	88.9% (16/18)	>0.999
NPV	95.3% (81/85)	91.2% (62/68)	0.487	96.2% (51/53)	90.0% (36/40)	0.433	93.8% (30/32)	92.9% (26/28)	>0.999
PPV	68.3% (43/63)	51.3% (41/80)	0.042	75.0% (27/36)	51.0% (25/49)	0.044	59.3% (16/27)	51.6% (16/31)	0.749

*NGS, next generation sequencing; BF, blastocoel fluid; SCM, spent culture medium; NPV, negative predictive value; PPV, positive predictive value.*

Our subgroup analysis of 89 triads of ICM-SCM-BF, which included 60 euploid blastocysts and 29 aneuploid blastocysts, showed a specificity of 85.0% for SCM and 60.0% for BF (*P* = 0.004) (Table 1) and a sensitivity of 93.1% for SCM and 86.2% for BF (*P* = 0.670). The NPV was 96.2% for SCM and 90.0% for BF (*P* = 0.443), and the PPV was 75.0% for SCM and 51.0% for BF (*P* = 0.044). The concordance rates of SCM and BF were 87.6% (78/89) and 69.7% (62/89), respectively, in this subgroup. Furthermore, our subgroup analysis of 59 triads of WB-SCM-BF samples revealed a specificity of 73.2% for SCM and 63.4% for BF (*P* = 0.476) and a sensitivity of 88.9% for both SCM and BF (*P* > 0.999). The NPVs were comparable between SCM and BF (93.8 versus 92.9%, *P* > 0.999). There was also no significant difference in PPV between SCM and BF (59.3 versus 51.6%, *P* = 0.749). The concordance rates of SCM and BF were 78.0% (46/59) and 69.5% (41/59), respectively, in this subgroup. Among 64 pairs of WB-SCM, the specificity of SCM was 81.0% (34/42), and the sensitivity was 95.5% (21/22) with an NPV of 97.1% and a PPV of 72.4% (Supplementary Table 1).

### Blastocoel Fluid and Spent Culture Medium From *in vitro* Fertilization Versus Intracytoplasmic Sperm Injection Embryos

Ninety-six blastocysts were obtained *via* IVF. Compared with the ICM/WB results, the BF and SCM had an overall ploidy concordance of 68.3% (43/63) and 84.4% (81/96), respectively, and sex concordance rates of 84.1% (53/63) and 99.0% (95/96), respectively. One hundred sixteen blastocysts were fertilized by ICSI. The overall ploidy concordance was 84.5% for SCM and 70.6% for BF, while the sex concordance rates of SCM and BF were similar (97.4 versus 91.8%) (Table 2). The overall ploidy concordance in IVF and ICSI was comparable for SCM (84.4 versus 84.5%, *P* = 0.983) and BF (68.3 versus 70.6%, *P* = 0.760).

**TABLE 2:** Chromosome ploidy and sex consistency in different fertilized embryos.

Insemination	Assay	Overall ploidy concordance	Sex concordance
IVF (*n* = 96)	BF (*n* = 63)	68.3% (43/63)	84.1% (53/63)
	SCM (*n* = 96)	84.4% (81/96)	99.0% (95/96)
ICSI (*n* = 116)	BF (*n* = 85)	70.6% (60/85)	91.8% (78/85)
	SCM (*n* = 116)	84.5% (98/116)	97.4% (113/116)

## Discussion

The current study showed that compared to BF samples, SCM samples yielded a more than 5-fold increase in DNA content prior to WGA, invariably provided reliable NGS data for comprehensive chromosome analysis and exhibited a significantly higher concordance rate with trophectoderm biopsy and ICM. The present study validated and further expanded our previous study on the NICS method using SCM for predicting the embryonic karyotype (Xu et al., 2016), showing that SCM samples offer a more effective source of embryo DNA for pre-implantation genetic screening.

In the current study, reliable NGS data were available in all 100% of the SCM samples but only in 69.8% of the BF samples. Thirty-eight BF samples failed to yield valid NGS data due to technical reasons, and 26 BF samples failed due to low reads because of low cfDNA content. This study showed that SCM samples yielded a more than 5-fold higher DNA content prior to WGA and a 2.8-fold higher DNA content following WGA than BF samples, which may be due mainly to incomplete aspiration of BF by blastocentesis. Currently, blastocentesis is still plagued by the technical issue of incomplete BF aspiration, as BF collection requires not only blastocyst expansion but also aspiration of the BF. A lower DNA level in diluted BF samples may lead to false positive results and hence a lower PPV due to uneven amplification and allele dropout (Huang et al., 2015; Hammond et al., 2016). Another reason behind the high proportion of false positive results with BF DNA is that the aneuploid cells may be excluded from the embryo and released in the BF during blastocyst formation, leaving a euploid ICM posited as embryo normalization (Tobler et al., 2015).

It has been proposed that DNA may be released into the BF and SCM from cells of the ICM and the trophectoderm during blastocyst development due to cell lysis, apoptosis or shedding of cellular debris. Previous studies aiming to test whether the BF can serve as an alternative for PGT-A have compared the array comparative genomic hybridization results of BF with polar body, blastomere or trophectoderm biopsy. Gianaroli et al. (2014) demonstrated a concordance of 95% for the ploidy condition of BF samples with either polar body or blastomere biopsy and a 97% concordance with trophectoderm biopsy. A consistently high concordance with trophectoderm was also reported in a subsequent study by the same group (Magli et al., 2016). However, Tobler et al. (2015) compared the results obtained from BF with those from the remaining embryos and observed a concordance rate of 48% for the karyotype and a concordance rate of 62% for the ploidy condition. In the present study, we observed concordant ploidy conditions with the ICM in 69.6% of BF samples, which is consistent with the results of Tobler et al. (2015). The high concordance observed by Gianaroli et al. (2014) and Magli et al. (2016) might be attributed to different cohorts of embryos in their studies. Specifically, embryos with a high aneuploid rate (74 and 84%) resulted in high concordance of BF with embryos. In contrast, embryos with a low aneuploid rate [31.7% in our study and 28% in the study by Tobler et al. (2015)] tend to produce a higher false positive rate with BF samples. Nevertheless, blastocentesis is an invasive procedure, although it is less invasive than embryo biopsy. In contrast, SCM is almost non-invasive for liquid biopsy is quite feasible and has a 100% rate of yielding reliable NGS data for subsequent analysis. Therefore, the use of BF as a single source of DNA is not recommended for pre-implantation genetic screening by PGT-A, especially in the cohort of embryos with potential for a high euploid rate.

DNA contamination remains a risk in the use of SCM as a source of DNA. The potential sources of contaminants include HSA, maternal DNA from cumulus cells (Hammond et al., 2016) and paternal DNA from sperm (for IVF embryos). However, in the present study, the blank media with/without HSA that were cultured under identical circumstances as embryos and hand no amplifiable DNA could yield valid NGS data, suggesting an extremely low likelihood of background contamination using SCM as a source of DNA. Vera-Rodriguez et al. (2018) demonstrated a mixed origin of cfDNA in SCM with maternal contamination, which in turn contributes to a high discordance (67%) of euploid conditions with trophectoderm biopsies. However, in the present study, we obtained 98.1% concordance on the sex chromosomes for SCM, suggesting the limited impact of maternal contamination on the assay results. In embryos conceived *via* IVF, free DNA released by sperm attached to the embryos is supposed to interfere with the accuracy of liquid biopsy-based testing. However, the results of the current study suggested that SCM and BF had comparable concordance rates in IVF and ICSI embryos. Previous studies suggested that the BF had a lower risk of contamination from cumulus cells, sperm and polar bodies that persisted to the blast stage than the SCM (Hammond et al., 2016). In contrast, compared with ICSI embryos, the accuracy of SCM, especially the sex consistency of IVF embryos, was similar to that of ICSI embryos, and the performance was better than that of BF samples, indicating that interference by cfDNA released by sperm was limited when using thawed blastocysts. If future studies show that IVF embryo SCM is not interfered with by granulosa cells or sperm, non-invasive PGT-A could be expanded to all IVF embryo transfer (ET) procedures.

Clinically, embryos are generally screened for transfers according to their morphological scores and through invasive PGT-A. Based on an NPV > 95% using SCM samples, we propose an embryo selection model involving morphological assessment followed by a non-invasive comprehensive chromosome screening by using SCM. Considering the low PPV value, if no euploid embryo is obtained after non-invasive assessment, a scoring system based on probability of ploidy abnormalities could be considered to prioritize embryos for transfer to prevent false exclusion of transferable embryos. We have developed an AI system based on machine learning, which may be ideal to optimize the selection of a single embryo for transfer, thus maximizing the chance of live birth and avoiding the waste of potentially qualified embryos. The articles are already being submitted (Chen et al., 2021).

Studies show that non-invasive PGT-A (niPGT-A) is prone to generating false positives depending on the maternal age of the population involved in the study. In other words, false positives will have less impact on the euploid/aneuploid concordance rate when the maternal age is higher, so we will perform large-scale RCTs for people over 35 years old who have already registered internationally.

However, there are still some limitations. First, to successfully isolate ICM from blastocysts, we studied fully re-expanded blastocysts with good morphological features, while blastocysts with poor grade scores were not analyzed. Second, there was a high euploid blastocyst rate in this study, and the aneuploid rate was low (31.8%), which may limit the strength of the analysis for aneuploid concordance; accordingly, the evidence was not as strong when the concern was aneuploidy. Third, most of the clinically collected samples are fresh samples, and it is not a routine operation to collect culture medium by cryo-resuscitation unless some suitable application scenarios cannot be directly applied to routine clinical fresh samples. For example, for couples with a history of transplantation failure or abortion, specialists can try to test the culture medium before ET to increase the probability of pregnancy, reduce the likelihood of abortion caused by chromosome abnormality, save time and limit the cost of pregnancy. Fourth, contamination occurs in the SCM (both paternal and maternal). In our datasets, we observed only two false negatives (Supplementary Table 2). False negatives are thought to be partially attributed to maternal contamination, such as cumulus cells, which have a balanced chromosomal content. This phenomenon exists in the culture medium samples (Feichtinger et al., 2017).

However, the low proportion of maternal contamination and higher concordance rate observed in the present study might be attributed to two factors: (a) our use of ICM instead of TE biopsy as the gold standard for comparison and (b) our use of thawed blastocysts. Whether cryopreservation facilitates diminishing the adhering paternal structures (e.g., cumulus cells and sperm) and decreases paternal contamination needs further investigation.

In the future, a standardized embryo culture and sample collection combining downstream detection technology and matched software should be built to predict euploidy before transplantation and to provide patients with the most suitable and most economical testing program, ultimately saving patients the time required for pregnancy and improving the overall success rate of IVF-ET.

## Conclusion

This is the first study to evaluate the chromosome constitution of BF or SCM for embryo liquid biopsy regarding the ICM as a gold standard. Our results suggest that compared with BF, SCM has a better diagnostic performance and is non-invasive for embryos. SCM might be a safer alternative material in embryo screening of clinical embryo transfer.

## Data Availability Statement

According to national legislation/guidelines, specifically the Administrative Regulations of the People’s Republic of China on Human Genetic Resources (http://www.gov.cn/zhengce/content/2019-06/10/content_5398829.htm, http://english.www.gov.cn/policies/latest_releases/2019/06/10/content_281476708945462.htm), no additional raw data is available at this time. Data of this project can be accessed after an approval application to the China National Genebank (CNGB, https://db.cngb.org/cnsa/). Please refer to https://db.cngb.org/, or email: CNGBdb@cngb.org for detailed application guidance. The accession code CNP0002305 should be included in the application.

## Ethics Statement

The studies involving human participants were reviewed and approved by the Ethics Committee of Northwest Women’s and Children’s Hospital, Xi’an, China (No. 2017121101). The patients/participants provided their written informed consent to participate in this study.

## Author Contributions

WS and JS designed the experiments and drafted the manuscript. WS and ZZ contributed to the clinical samples. DW, YY, XX, and JW performed the experiments and analyzed the data. WS, SL, and QL reviewed the manuscript. The work was finalized by JS with the assistance of all the authors. All authors contributed to the article and approved the submitted version.

## Conflict of Interest

YY, JW, and SL were employed by Yikon Genomics Company, Ltd. The remaining authors declare that the research was conducted in the absence of any commercial or financial relationships that could be construed as a potential conflict of interest.

## Publisher’s Note

All claims expressed in this article are solely those of the authors and do not necessarily represent those of their affiliated organizations, or those of the publisher, the editors and the reviewers. Any product that may be evaluated in this article, or claim that may be made by its manufacturer, is not guaranteed or endorsed by the publisher.

## Footnotes

1
http://www.chictr.org.cn

## References

[B1] Campos-Galindo I. Garcia-Herrero S. Martinez-Conejero J. A. Ferro J. Simon C. Rubio C. (2015). Molecular analysis of products of conception obtained by hysteroembryoscopy from infertile couples. *J. Assist. Reprod. Genet.* 32 839–848. 10.1007/s10815-015-0460-z 25779005 PMC4429442

[B2] Capalbo A. Wright G. Elliott T. Ubaldi F. M. Rienzi L. Nagy Z. P. (2013). FISH reanalysis of inner cell mass and trophectoderm samples of previously array-CGH screened blastocysts shows high accuracy of diagnosis and no major diagnostic impact of mosaicism at the blastocyst stage. *Hum. Reprod.* 28 2298–2307. 10.1093/humrep/det245 23739221

[B3] Chen L. Li W. Liu Y. Peng Z. Cai L. Zhang N. (2021). *Machine Learning-Guided Noninvasive Embryo Selection for Clinical in Vitro Fertilization Treatment to Avoid Wasting Potentially Qualified Embryos.* Nanjing: ResearchGate. 10.21203/rs.3.rs-617438/v1

[B4] Fang R. Yang W. Zhao X. Xiong F. Guo C. Xiao J. (2019). Chromosome screening using culture medium of embryos fertilised in vitro: a pilot clinical study. *J. Transl. Med.* 17:73. 10.1186/s12967-019-1827-1 30849973 PMC6408780

[B5] Feichtinger M. Vaccari E. Carli L. Wallner E. Mädel U. Figl K. (2017). Non-invasive preimplantation genetic screening using array comparative genomic hybridization on spent culture media: a proof-of-concept pilot study. *Reproductive Biomed. Online* 34 583–589. 10.1016/j.rbmo.2017.03.015 28416168

[B6] Fragouli E. Wells D. (2012). Aneuploidy screening for embryo selection. *Sem. Reproductive Med.* 30 289–301. 10.1055/s-0032-1313908 22723010

[B7] Gianaroli L. Magli M. C. Pomante A. Crivello A. M. Cafueri G. Valerio M. (2014). Blastocentesis: a source of DNA for preimplantation genetic testing. Results from a pilot study. *Fertil Steril.* 102 1692–1699.e6. 10.1016/j.fertnstert.2014.08.021. 25256935

[B8] Hammond E. R. Shelling A. N. Cree L. M. (2016). Nuclear and mitochondrial DNA in blastocoele fluid and embryo culture medium: evidence and potential clinical use. *Hum. Reprod.* 31 1653–1661. 10.1093/humrep/dew132 27270971

[B9] Huang L. Ma F. Chapman A. Lu S. Xie X. S. (2015). Single-cell whole-genome amplification and sequencing: methodology and applications. *Annu. Rev. Genomics Hum. Genet.* 16 79–102. 10.1146/annurev-genom-090413-25352 26077818

[B10] Kuznyetsov V. Madjunkova S. Antes R. Abramov R. Motamedi G. Ibarrientos Z. (2018). Evaluation of a novel non-invasive preimplantation genetic screening approach. *PLoS One* 13:e0197262. 10.1371/journal.pone.0197262 29746572 PMC5944986

[B11] Magli M. C. Albanese C. Crippa A. Tabanelli C. Ferraretti A. P. Gianaroli L. (2019). Deoxyribonucleic acid detection in blastocoelic fluid: a new predictor of embryo ploidy and viable pregnancy. *Fertil. Steril.* 111 77–85. 10.1016/j.fertnstert.2018.09.016 30528055

[B12] Magli M. C. Pomante A. Cafueri G. Valerio M. Crippa A. Ferraretti A. P. (2016). Preimplantation genetic testing: polar bodies, blastomeres, trophectoderm cells, or blastocoelic fluid? *Fertil Steril.* 105 676–683.e5. 10.1016/j.fertnstert.2015.11.018. 26658131

[B13] Mastenbroek S. Twisk M. Van der Veen F. Repping S. (2011). Preimplantation genetic screening: a systematic review and meta-analysis of RCTs. *Hum. Reprod. Update* 17 454–466. 10.1093/humupd/dmr003 21531751

[B14] Palini S. Galluzzi L. De Stefani S. Bianchi M. Wells D. Magnani M. (2013). Genomic DNA in human blastocoele fluid. *Reprod. Biomed. Online* 26 603–610. 10.1016/j.rbmo.2013.02.012 23557766

[B15] Rubio C. Pehlivan T. Rodrigo L. Simon C. Remohi J. Pellicer A. (2005). Embryo aneuploidy screening for unexplained recurrent miscarriage: a minireview. *Am. J. Reprod. Immunol.* 53 159–165. 10.1111/j.1600-0897.2005.00260.x 15760376

[B16] Rubio C. Rienzi L. Navarro-Sanchez L. Cimadomo D. Garcia-Pascual C. M. Albricci L. (2019). Embryonic cell-free DNA versus trophectoderm biopsy for aneuploidy testing: concordance rate and clinical implications. *Fertil. Steril.* 112 510–519. 10.1016/j.fertnstert.2019.04.038 31200971

[B17] Shamonki M. I. Jin H. Haimowitz Z. Liu L. (2016). Proof of concept: preimplantation genetic screening without embryo biopsy through analysis of cell-free DNA in spent embryo culture media. *Fertil. Steril.* 106 1312–1318. 10.1016/j.fertnstert.2016.07.1112 27565258

[B18] Shang W. Zhang Y. Shu M. Wang W. Ren L. Chen F. (2018). Comprehensive chromosomal and mitochondrial copy number profiling in human IVF embryos. *Reprod. Biomed. Online* 36 67–74. 10.1016/j.rbmo.2017.10.110 29203383

[B19] Sullivan-Pyke C. Dokras A. (2018). Preimplantation genetic screening and preimplantation genetic diagnosis. *Obstet. Gynecol. Clin. North Am.* 45 113–125. 10.1016/j.ogc.2017.10.009 29428279

[B20] Tobler K. J. Zhao Y. Ross R. Benner A. T. Xu X. Du L. (2015). Blastocoel fluid from differentiated blastocysts harbors embryonic genomic material capable of a whole-genome deoxyribonucleic acid amplification and comprehensive chromosome microarray analysis. *Fertil. Steril.* 104 418–425. 10.1016/j.fertnstert.2015.04.028 26006737

[B21] Vera-Rodriguez M. Diez-Juan A. Jimenez-Almazan J. Martinez S. Navarro R. Peinado V. (2018). Origin and composition of cell-free DNA in spent medium from human embryo culture during preimplantation development. *Hum. Reprod.* 33 745–756. 10.1093/humrep/dey028 29471395

[B22] Wu Y. Lv Z. Yang Y. Dong G. Yu Y. Cui Y. (2014). Blastomere biopsy influences epigenetic reprogramming during early embryo development, which impacts neural development and function in resulting mice. *Cell Mol. Life. Sci.* 71 1761–1774. 10.1007/s00018-013-1466-1462 24037382 PMC11114061

[B23] Xu J. Fang R. Chen L. Chen D. Xiao J. P. Yang W. (2016). Noninvasive chromosome screening of human embryos by genome sequencing of embryo culture medium for in vitro fertilization. *Proc. Natl. Acad. Sci. U S A.* 113 11907–11912. 10.1073/pnas.1613294113 27688762 PMC5081593

[B24] Yeung Q. S. Y. Zhang Y. X. Chung J. P. W. Lui W. T. Kwok Y. K. Y. Gui B. (2019). A prospective study of non-invasive preimplantation genetic testing for aneuploidies (NiPGT-A) using next-generation sequencing (NGS) on spent culture media (SCM). *J. Assist. Reprod. Genet.* 36 1609–1621. 10.1007/s10815-019-01517-1517 31292818 PMC6707994

[B25] Zeng Y. Lv Z. Gu L. Wang L. Zhou Z. Zhu H. (2013). Preimplantation genetic diagnosis (PGD) influences adrenal development and response to cold stress in resulting mice. *Cell Tissue Res.* 354 729–741. 10.1007/s00441-013-1728-1721 24104561

[B26] Zhang Y. Li N. Wang L. Sun H. Ma M. Wang H. (2016). Molecular analysis of DNA in blastocoele fluid using next-generation sequencing. *J. Assist. Reprod. Genet.* 33 637–645. 10.1007/s10815-016-0667-667 26899834 PMC4870435

