# Supplementary material for: Ploidy Testing of Blastocoel Fluid for Screening May Be Technically Challenging and More Invasive Than That of Spent Cell Culture Media
Source: Front Physiol. 2022 Feb 21;13:794210. doi: 10.3389/fphys.2022.794210 (PMC8900197; doi:10.3389/fphys.2022.794210)
Supplement: Supplementary file 1 [file Data_Sheet_1.docx]

**Supplemental Table 1.** Performance of the remaining SCM relative to WB in detecting aneuploidy.

| **Assay** | **Specificity** | **Sensitivity** | **NPV** | **PPV** |
| --- | --- | --- | --- | --- |
| **SCM (n=64)** | 81.0% (34/42) | 95.5% (21/22) | 97.1% (34/35) | 72.4% (21/29) |

**Supplemental Table 2.**

**Supplemental Table 2-1.** Comparison of karyotype in spent culture medium (SCM) or blastocoel fluid (BF) with that of the inner cell mass (ICM).

| E No. | | Insemination | ICM | SCM | BF |
| --- | --- | --- | --- | --- | --- |
| 8 | | ICSI | 45,XX,-22 | 46,XX,+20,-22 | 51,XX,-Xmos,-1,+2,+5,+6,+9,+12,-14,+18,+2 ,+21,-22 |
| 12 | | ICSI | 46,XY,+12mos,+13mos | 46,XY,+Xmos,-Ymos | 48,XY,+1,+3 |
| 13 | | ICSI | 47,XY,+14 | 48,XY,+5,+14 | 47,XY,+14 |
| 19 | | ICSI | 46,XY,-Ymos | 47,XX,+17 | 46,XY,-Ymos |
| 33 | | ICSI | 47,XX,+16 | 47,XX,+16 | 47,XY,+Xmos,-Ymos,+16 |
| 40 | | ICSI | 45,XY,-7 | 45,XY,-7 | 45,XY,-7 |
| 41 | | ICSI | 45,XY,-22 | 45,XY,-22 | 49,XY,+3,+8,+20 |
| 46 | | ICSI | 45,XY,-22 | 45,XY,-22 | 46,XY,-22mos |
| 55 | | ICSI | 45,XY,-21,+22mos | 46,XY,-21,+22 | 46,XY,-21mos,+22mos |
| 58 | | ICSI | 47,XY,+22 | 47,XY,+22 | 47,XY,+22 |
| 65 | | ICSI | 47,XX,+21 | 45,XX,+21 | 47,XX,+21,+22mos |
| 88 | | ICSI | 48,XY,+14,+15 | 47,XY,+19 | 47,XY,+14mos,+15 |
| 94 | | ICSI | 47,XX,+16 | 47,XX,+16 | 47,XX,+16 |
| 96 | | ICSI | 47,XX,+2 | 46,XX,-10mos,+12mos,-22mos | 46,XX,+2mos |
| 115 | | ICSI | 45,XY,-15 | 45,XY,-15 | 45,XY,-15 |
| 128 | | ICSI | 45,XX,-22 | 45,XX,-22 | 45,XX,-22 |
| 139 | | ICSI | 45,XY,-16 | 45,XY,-16 | 45,XY,-16 |
| 164 | | ICSI | 47,XX,+14 | 47,XX,+14 | 47,XX,+14 |
| 171 | | ICSI | 45,XX,-8 | 45,XX,-8 | 45,XX,-8 |
| 172 | | ICSI | 45,XY,-21 | 45,XY,-21 | 46,XY,-21mos |
| 175 | | ICSI | 46,XY,+14mos | 47,XY,+14 | 47,XY,+14 |
| 195 | | ICSI | 45,XX,-10 | 45,XY,-Ymos,-10 | 46,XX,-Xmos,+9mos,-10mos,+16mos,+20mos |
| 196 | | ICSI | 47,XX,+21 | 47,XX,+21 | 47,XX,+21 |
| 201 | | ICSI | 47,XX,+14 | 47,XX,+14 | 47,XX,+14 |
| 205 | | ICSI | 45,XY,-13 | 45,XY,-13 | 45,XY,-13 |
| 24 | | ICSI | 50XX,+2,+4,+17,+18 | 46,XX | 46,XX |
| 35 | | ICSI | 45,XY,-21 | 45,XY,-21 | 46,XY |
| 37 | | ICSI | 46,XX,-15mos | 46,XX | 46,XX |
| 215 | | ICSI | 45,X,-X | 47,XXX,+X | 46,XX |
| **ICM euploid embryos (N=60)** | | | | | |
| **Euploid concordant (n=31)** | | | | | |
| **E No.** | **Insemination** | | **ICM** | **SCM** | **BF** |
| 1 | IVF | | 46,XY | 46,XY | 46,XY |
| 7 | ICSI | | 46,XY | 46,XY | 46,XY |
| 26 | ICSI | | 46,XX | 46,XX | 46,*XY* |
| 45 | IVF | | 46,XY | 46 XY | 46,XY |
| 57 | ICSI | | 46,XY | 46,XY | 46,XY |
| 59 | ICSI | | 46,XY | 46,XY | 46,XY |
| 63 | ICSI | | 46,XY | 46,XY | 46,XY |
| 67 | ICSI | | 46,XY | 46,XY | 46,XY |
| 72 | IVF | | 46,XY | 46,XY | 46,XY |
| 74 | IVF | | 46,XY | 46,XY | 46,XY |
| 78 | IVF | | 46,XX | 46,XX | 46,XX |
| 86 | IVF | | 46,XY | 46,XY | 46,XY |
| 87 | IVF | | 46,XY | 46,XY | 46,XY |
| 90 | IVF | | 46,XX | 46,XX | 46,XX |
| 91 | IVF | | 46,XY | 46,XY | 46,XY |
| 105 | IVF | | 46,XY | 46,XY | 46,XY |
| 109 | IVF | | 46,XX | 46,XX | 46,XX |
| 112 | IVF | | 46,XY | 46 XY | 46,XY |
| 118 | IVF | | 46,XY | 46,XY | 46,XY |
| 131 | IVF | | 46,XY | 46,XY | 46,XY |
| 132 | IVF | | 46,XX | 46,XX | 46,XX |
| 133 | IVF | | 46,XY | 46,XY | 46,XY |
| 140 | IVF | | 46,XY | 46,XY | 46,XY |
| 151 | ICSI | | 46,XX | 46,XX | 46,XX |
| 165 | IVF | | 46,XY | 46,XY | 46,XY |
| 181 | IVF | | 46,XY | 46,XY | 46,XY |
| 193 | IVF | | 46,XY | 46,XX | 46,XY |
| 198 | ICSI | | 46,XY | 46,XY | 46,XY |
| 199 | ICSI | | 46,XY | 46,XY | 46,XY |
| 202 | IVF | | 46,XY | 46,XY | 46,XY |
| 203 | IVF | | 46,XY | 46,XY | 46,XY |
| 207 | ICSI | | 46,XY | 46,XY | 46,XY |
| **SCM-BF-aneuploid (n=5)** | | | | | |
| **E No.** | **Insemination** | | **ICM** | **SCM** | **BF** |
| 18 | ICSI | | 46,XY | 48,XY,+14,+19 | 53,XY,+Xmos,+2,+3,+12,+14,+15,+19,+20 |
| 38 | ICSI | | 46,XX | 46,XX,+4mos | 46,XX,+Xmos,+4,-5,-9,-10,+14 |
| 81 | IVF | | 46,XX | 45,XX,-8 | 48,XX,-8,+9,+14,-15,+20,+21 |
| 174 | ICSI | | 46,XX | 47,XX,+16 | 47,XXY,-Ymos |
| 183 | ICSI | | 46,XY | 45,XY,-12 | 46,XY,+7,-12 |
| **BF-aneuploid (n=19)** | | | | | |
| **E No.** | **Insemination** | | **ICM** | **SCM** | **BF** |
| 11 | ICSI | | 46,XY | 46,XY | 46,XY,+1,-4 |
| 16 | ICSI | | 46,XX | 46,XX | 48,X,-X,-4,+6,+9,+18,+22 |
| 17 | ICSI | | 46,XY | 46,XY | 45,XY,-1 |
| 23 | ICSI | | 46,XY | 46,XY | 48,XY,+17,+19 |
| 27 | ICSI | | 46,XY | 46,XY | 51,XY,-Xmos,+5,+11,+13,+14,+16 |
| 32 | ICSI | | 46,XX | 46,XX | 49,XY,-Ymos +15,+16,+18 |
| 50 | ICSI | | 46,XY | 46,XY | 56,XXY,+X,+1,+3,+7,+8,+14,+17,+18,+19,+20 |
| 75 | IVF | | 46,XX | 46,XX | 49,XY,+Xmos,-Ymos,-4,+6,+10,+12,+18 |
| 89 | IVF | | 46,XX | 46,XX | 50,XX,+8,+12,+18,+21 |
| 95 | IVF | | 46,XY | 46,XY | 45,XY,-3 |
| 101 | IVF | | 46,XX | 46,XX | 47,XY,+Xmos,-Ymos,+17 |
| 104 | IVF | | 46,XX | 46,XX | 48,XY,-Ymos,+8,+19 |
| 113 | IVF | | 46,XY | 46,XY | 47,XY,+14 |
| 123 | IVF | | 46,XX | 46,XX | 50,XY,+Xmos,-Ymos,+1,+6,+13,+17 |
| 127 | IVF | | 46,XX | 46,XX | 46,XY,+Xmos,-Ymos |
| 137 | IVF | | 46,XY | 46,XY | 48,XY,+Xmos,+Ymos,-9,+11,+19,+21 |
| 138 | IVF | | 46,XX | 46,XX | 46,XY,+Xmos,-Ymos |
| 182 | ICSI | | 46,XX | 46,XX | 47,XY,+Xmos,-Ymos,+14 |
| 200 | IVF | | 46,XX | 46,XX | 46,XY,+Xmos,-Ymos |
| **SCM-aneuploid (n=4)** | | | | | |
| **E No.** | **Insemination** | | **ICM** | **SCM** | **BF** |
| 9 | ICSI | | 46,XY | 47,XY,+7 | 46,XY |
| 125 | IVF | | 46,XY | 53XXY,+8,+9,+13,+15,+17,+19 | 46,XY |
| 180 | IVF | | 46,XY | 48,XY,+2,+12,-19,+20 | 46,XY |
| 211 | ICSI | | 46,XY | 47,XY,+Xmos,+22 | 46,XY |

**Supplemental Table 2-2.** Comparison of karyotype in spent culture medium (SCM) or blastocoel fluid (BF) with that in the whole embryo.

| **Whole aneuploid/mosaic embryos (N=18)** | | | | |
| --- | --- | --- | --- | --- |
| **NO.** | **Insemination** | **SCM** | **BF** | **Whole embryo** |
| 14 | ICSI | 47,XX,+13 | 47,XX,+13 | 47,XX,+13 |
| 15 | ICSI | 46,XY | 46,XY | 46,XY,+4,-13 |
| 20 | ICSI | 46,XY | 46,XY | 46,XY,+7mos |
| 39 | ICSI | 48,XY,+1,+16 | 48,XY,-Xmos,+1,+8 | 48,XY,+1,+16 |
| 48 | ICSI | 48,XY,+9,+15 | 48,XY,+9,+12 | 46,XY,+9,-15 |
| 51 | ICSI | 45,XY,-14 | 50,XY,+4,+9,+14,+20 | 46,XY,-14 |
| 130 | IVF | 45,X,-X | 47,XY,+Xmos,+14 | 46,XX,-Xmos |
| 134 | IVF | 47,XY,+15 | 46,XY,+Xmos | 47,XY,+15 |
| 141 | ICSI | 47,XY,+19 | 47,XY,+19 | 47,XY,+19 |
| 145 | ICSI | 45,XY,-13 | 45,XY,-13 | 45,XY,-13 |
| 173 | ICSI | 44,XY,-17,-18 | 46,XY,-17mos,-18mos | 44,XXY,-17,-18 |
| 176 | ICSI | 48,XX,+1,-7,-8,+13,+14,+18 | 45,XX,-8 | 45,XX,-8 |
| 177 | ICSI | 40,XY,-Ymos,-9,-13,-16,-18,-21,-22 | 48,XY,+1,+16 | 48,XY,+1,+16 |
| 191 | ICSI | 47,XX,+5 | 44,XX,-5,-13,-14mos,+15mos,-Xmos | 46,XX,+5mos |
| 197 | IVF | 45,XX,-16 | 45,XX,-16 | 45,XX,-16 |
| 204 | ICSI | 45,XY,-21 | XY,-6,+13mos,-18mos,-21 | 45,XY,-21 |
| 206 | ICSI | 48,XX,+15 | 48,XX,+15 | 48,XX,+15 |
| 208 | ICSI | 45,XX,-14 | 46,XX,-14mos | 45,XX,-14 |
| **Whole euploid embryos (N=41)** | | | | |
| **NO.** | **Insemination** | **SCM** | **BF** | **Whole embryo** |
| 3 | ICSI | 47,XX,+8 | 47,XY,-Ymos,+15 | 46,XX |
| 4 | ICSI | 46,XY,+Xmos,-Ymos | 46,XY | 46,XY |
| 5 | IVF | 46,XY | 49,XY,+4,+14,+19 | 46,XY |
| 6 | IVF | 47,XY,+15 | 46,XY | 46,XY |
| 10 | ICSI | 46,XX | 49,XXX,+X,-1,+2,+3,-5,-6,+7,-9,+10,-11,+12,+13,+14,+20,-22 | 46,XX |
| 25 | ICSI | 46,XY | 46,XY | 46,XY |
| 28 | ICSI | 46,XY | 46,XY | 46,XY |
| 30 | ICSI | 47,XY,+5 | 47,XY,+22 | 46,XY |
| 36 | ICSI | 46,XX | 45,XY,+Xmos,-Ymos,-14(×1) | 46,XX |
| 44 | IVF | 46,XY,+Xmos,-Ymos | 46,XY | 46,XY |
| 53 | ICSI | 46,XY | 46,XY | 46,XY |
| 56 | ICSI | 46,XY | 46,XY | 46,XY |
| 61 | ICSI | 46,XX | 46,XY,+Xmos,-Ymos | 46,XX |
| 62 | ICSI | 47,XY,+16 | 46,XY | 46,XY |
| 69 | ICSI | 46,XY | 46,XY | 46,XY |
| 70 | ICSI | 46,XY | 46,XY | 46,XY |
| 73 | IVF | 46,XY | 46,XY | 46,XY |
| 77 | IVF | 46,XX | 46,XX | 46,XX |
| 79 | IVF | 46,XY | 46,XY | 46,XY |
| 80 | IVF | 46,XY | 49,XY,+6,+12,+15 | 46,XY |
| 84 | IVF | 46,XY,+2,+8,-17,-21 | 44,XY,-5,-17 | 46,XY |
| 85 | IVF | 46,XY | 48,XY,+6,+9 | 46,XY |
| 93 | IVF | 46,XX | 46,XX | 46,XX |
| 99 | IVF | 46,XX | 46,XX | 46,XX |
| 102 | IVF | 46,XX | 46,XX | 46,XX |
| 103 | IVF | 46,XX | 46,XX | 46,XX |
| 110 | IVF | 46,XX | 46,XX | 46,XX |
| 117 | IVF | 46,XY | 46,XY | 46,XY |
| 121 | IVF | 47,XY,-1,+14,+17 | 46,XY | 46,XY |
| 122 | IVF | 46,XX | 46,XY,+Xmos,-Ymos | 46,XX |
| 126 | IVF | 45,XX,-21 | 47,XY,+Xmos,-Ymos,+14 | 46,XX |
| 135 | IVF | 46,XY | 47,XY,+Xmos,+17 | 46,XY |
| 136 | IVF | 46,XY | 46,XY | 46,XY |
| 149 | ICSI | 46,XY,+Xmos | 46,XY | 46,XY |
| 153 | ICSI | 46,XY | 46,XY,-Y,mos,+1mos | 46,XY |
| 162 | IVF | 46,XY | 46,XY,+Xmos | 46,XY |
| 185 | IVF | 46,XY | 46,XY | 46,XY |
| 187 | ICSI | 46,XY | 46,XY | 46,XY |
| 188 | ICSI | 46,XY | 48,XY,+9,+11 | 46,XY |
| 194 | IVF | 46,XX | 46,XX | 46,XX |
| 209 | IVF | 47,XY,+Xmos,-Ymos,+19 | 46,XY | 46,XY |

**Supplemental Table 2-3.** Comparison of karyotype in spent culture medium (SCM) with that in the whole embryo.

| Whole aneuploid embryos (n=22) | | | |
| --- | --- | --- | --- |
| NO | **Insemination** | SCM | Whole embryo |
| 29 | ICSI | 47,XX,+14 | 47,XX,+14 |
| 31 | ICSI | 46,XX,+13,-14 | 46,XX,+13,-14 |
| 43 | ICSI | 45,XY,-22 | 45,XY,-22 |
| 52 | ICSI | 47,XX,+21 | 46,XX,+21mos |
| 54 | ICSI | 47,XX,-Xmos,+14 | 46,X,-X,+14 |
| 66 | ICSI | 46,XX,-3mos,+13,-21 | 45,XX,-3,+13,-21 |
| 68 | ICSI | 45,XY,-13 | 45,XY,-10mos,-13,+14mos |
| 76 | ICSI | 47,XY,+22 | 47,XY,+22 |
| 97 | ICSI | 46,XX,-18,+21 | 46,XX,-18,+21 |
| 108 | ICSI | 47,XX,+13 | 47,XX,+13 |
| 129 | IVF | 45,XY,-4 | 45,XY,-4 |
| 143 | ICSI | 48,XXY,+X,-Ymos,+16 | 46,XX,-15mos |
| 157 | ICSI | 46,XX,+22mos | 45,XX,-22 |
| 163 | IVF | 47,XX,+16 | 47,XX,+16 |
| 170 | IVF | 46,XX,-18mos | 45,XX,-18 |
| 178 | ICSI | 45,XX,-18 | 45,XX,-5 |
| 179 | ICSI | 45,XY,-16 | 45,XY,-16 |
| 190 | ICSI | 46,XY,+7mos | 46,XY,+7mos |
| 192 | ICSI | 47,XY,+Xmos,-2mos,-5mos,+16(×3) | 47,XY,+16 |
| 210 | ICSI | 45,XY,-16 | 45,XY,-16 |
| 212 | ICSI | 45,XX,-21 | 45,XX,-21 |
| 214 | ICSI | 46,XY | 46,XY,+Xmos,+Ymos |
| Euploid embryos (N=42) | | | |
| 2 | ICSI | 46,XX | 46,XX |
| 34 | ICSI | 46,XX | 46,XX |
| 42 | IVF | 47,XXY,+X,-Ymos | 46,XX |
| 47 | ICSI | 46,XX | 46,XX |
| 60 | ICSI | 46,XX | 46,XX |
| 64 | ICSI | 46,XX | 46,XX |
| 71 | ICSI | 46,XY | 46,XY |
| 82 | IVF | 46,XY | 46,XY |
| 83 | IVF | 46,XY | 46,XY |
| 92 | IVF | 46,XX | 46.XX |
| 98 | IVF | 46,XY | 46,XY |
| 100 | IVF | 46,XX | 46,XX |
| 106 | IVF | 46,XY | 46,XY |
| 107 | IVF | 46,XY | 46,XY |
| 111 | IVF | 46,XY | 46,XY |
| 114 | IVF | 46,XX | 46,XX |
| 116 | IVF | 46,XX | 46,XX |
| 119 | IVF | 46,XX,-Xmos | 46,XX |
| 120 | IVF | 46,XX | 46,XX |
| 124 | IVF | 46,XY | 46,XY |
| 142 | IVF | 46,XX | 46,XX |
| 144 | ICSI | 46,XY | 46,XY |
| 146 | ICSI | 47,XY,+18 | 46,XY |
| 147 | ICSI | 46,XX | 46,XX |
| 148 | ICSI | 46,XY | 46,XY |
| 150 | ICSI | 46,XY,-4,+20 | 46,XY |
| 152 | ICSI | 46,XY | 46,XY |
| 154 | IVF | 46,XX | 46,XX |
| 155 | IVF | 46,XX | 46,XX |
| 156 | IVF | 46,XX | 46,XX |
| 158 | IVF | 47,XX,+18 | 46,XX |
| 159 | IVF | 46,XX | 46,XX |
| 160 | IVF | 46,XY | 46,XY |
| 161 | IVF | 46,XX | 46,XX |
| 166 | IVF | 48,XX,-5,+12,+14,-15,+16,+21 | 46,XX |
| 167 | IVF | 46,XX | 46,XX |
| 168 | IVF | 46,XY | 46,XY |
| 169 | IVF | 45,XX,-4 | 46,XX |
| 184 | IVF | 46,XY | 46,XY |
| 186 | IVF | 46,XY,+Xmos,-Ymos | 46,XY |
| 189 | IVF | 46,XX | 46,XX |
| 213 | IVF | 46,XX | 46,XX |
