# Supplementary material for: Ploidy Testing of Blastocoel Fluid for Screening May Be Technically Challenging and More Invasive Than That of Spent Cell Culture Media
Source: Front Physiol. 2022 Feb 21;13:794210. doi: 10.3389/fphys.2022.794210 (PMC8900197; doi:10.3389/fphys.2022.794210)
Supplement: Supplementary file 2 [file Data_Sheet_2.docx]

**Supplemental Figures**

**
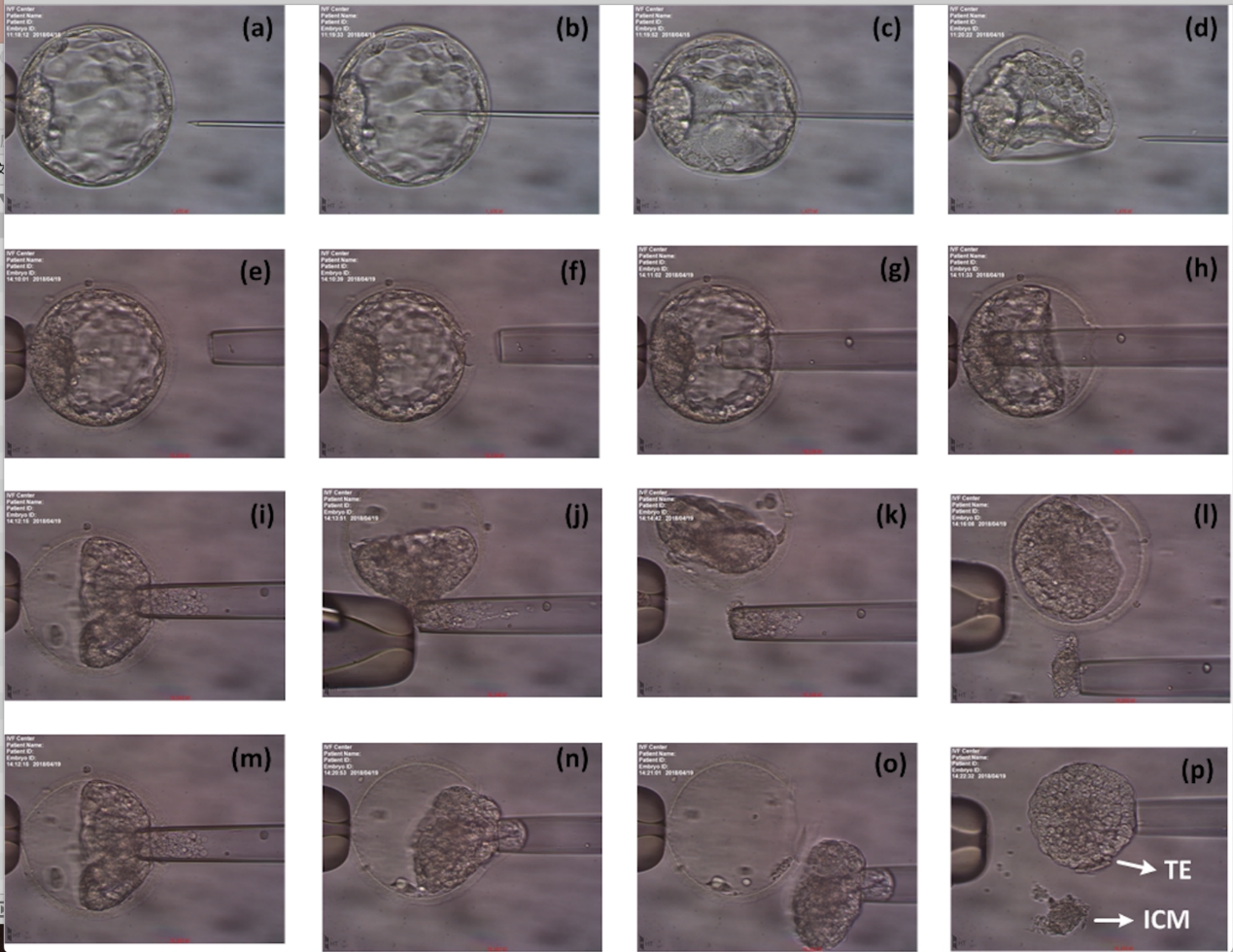
**

**Supplemental Figure S1.** Photographs of blastocentesis and blastocyst biopsy. (a-d): The blastocyst was immobilized with a holding pipette (a). An intracytoplasmic sperm injection pipette was inserted into the cavity from the point of contact between two trophectoderm cells to minimize the amount of crossed cytoplasm (b). The blastocyst fluid was gently aspirated to allow the blastocoel cavity to collapse around the needle while simultaneously withdrawing the needle (c-d). Attention was given to avoid the aspiration of cellular material and cell injury by the needle. Inner cell mass (ICM) biopsy (e-l): The blastocyst was secured from the ICM side with a holding pipette; then, with the assistance of a laser, a hole was made through the zona pellucida and trophectoderm layer (e). The biopsy pipette was directly introduced into the blastocoel cavity (f), pushing in and stretching the trophectoderm layer mechanically (h). The ICM was then aspirated and removed from the embryo with the assistance of a laser and mechanical cutting (i-j). The remaining trophectoderm tissue was stretched out of the zona pellucida (m-p). ICM: inner cell mass; TE: trophectoderm.


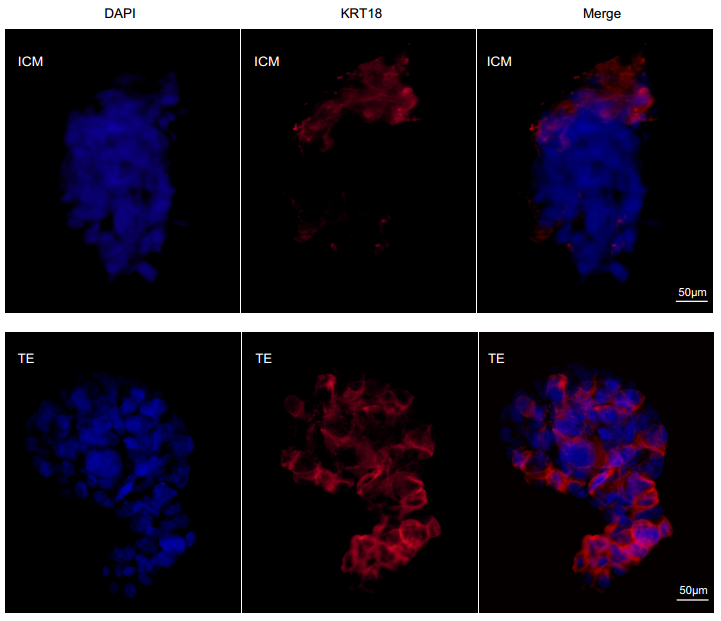


**Supplemental Figure S2.** A video of inner cell mass biopsy and fluorescence staining data of inner cell mass tissue are available at Human Reproduction online. Fluorescence microscopy imaging of the isolated ICM and the related trophectoderm obtained from a good-quality blastocyst subjected to ICM biopsy is shown (×40 magnification). Both tissues (isolated ICM and trophectoderm) were immunostained for KRT18, a highly specific trophectoderm biomarker (in red). The ICM sample shows only a few positive KRT18 cells, whereas the corresponding trophectoderm is totally positive for KRT18 immunostaining (middle panels). The right panels show merged images with DAPI staining (left panels, blue nuclei) for the same sections.


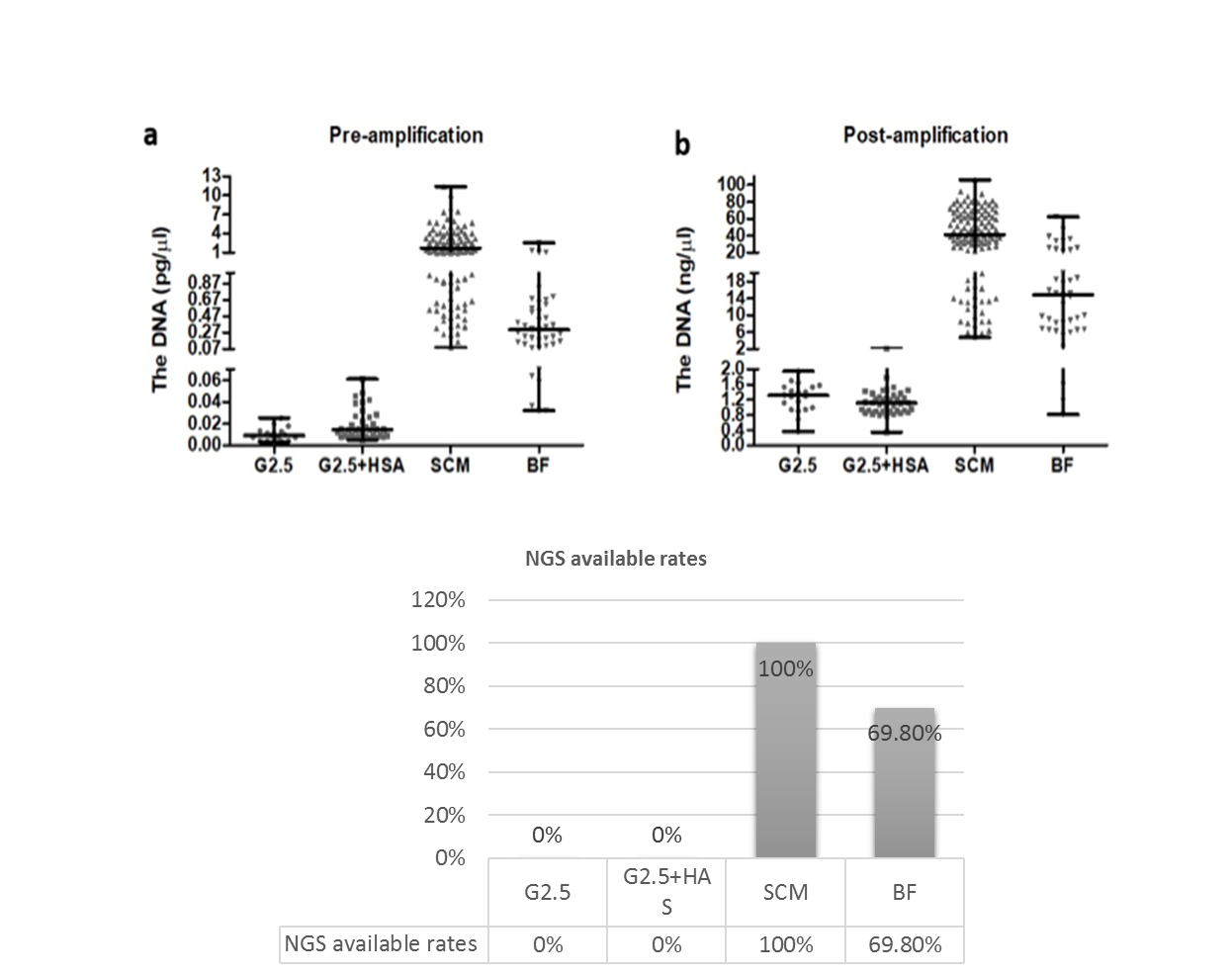


**Supplemental Figure S3. Cell-free DNA quantification in blastocyst fluid (BF), spent culture medium (SCM), and media controls.** (a-b) cfDNA quantification was performed before and after amplification in BF and SCM. In addition, the media control [(G2, G2+human serum albumin (HSA)] free of contact with embryos was investigated. (c) The positive rate is the proportion of the number of effective results after sequencing to the number of samples sequenced. DNA sequencing was routinely carried out in amplified samples, including all BF samples, SCM samples and blastocyst tissues. The media controls (5 cases of G2 and 10 cases of G2+HSA) with more than 1.5 ng/μL DNA after amplification were sequenced; however, no valid result was generated. *P < 0.05 **P < 0.01 ***P < 0.001.
